# Transformer-based coreference resolution modeling for Amharic text

**DOI:** 10.1038/s41598-026-46130-8

**Published:** 2026-06-12

**Authors:** Lingerew Bantie Asmare

**Affiliations:** Department of Information Science and Technology, School of Informatics and Artifi cial Intelligence, Mehal Meda Campus, Debre Berhan University, Debre Berhan, Ethiopia

**Keywords:** Amharic coreference resolution, Multilingual BERT, Low-resource NLP, Transformer, Mention-pair model, Word embeddings, Natural language processing, Engineering, Mathematics and computing

## Abstract

Coreference resolution is the task of identifying all textual expressions that refer to the same real-world entity within a document. Coreference resolution is essential for several NLP applications, including machine translation, question answering, information extraction, summarization, and dialogue systems. While substantial progress has been made in high-resource languages such as English and Arabic. Amharic is a morphologically rich, low-resource language remains underexplored due to the scarcity of annotated datasets and specialized language tools. This study proposes a transformer-based approach for Amharic coreference resolution using multilingual BERT (mBERT**)**. The proposed system combines contextual embeddings extracted from mBERT with manually engineered mention-pair features, such as sentence-level distance and exact string matching, through a supervised mention-pair classification framework. The system is composed of two main phases: training and testing. The training phase includes preprocessing, tokenization, sentence segmentation, feature extraction, and model training. The testing phase involves similar preprocessing steps and the prediction of coreference clusters. We created a novel annotated Amharic corpus consisting of 312 documents, 5428 sentences, 18,763 mentions, and 4912 coreference clusters to train and evaluate the model. Standard coreference evaluation metrics, including MUC, B^3^, CEAF-m, CEAF-e, and BLANC, were employed. The results show that the proposed model outperforms existing state-of-the-art Amharic systems, achieving F1-scores of 80%, 85.71%, 90.9%, 88.86%, and 81.7% across the respective metrics. This study demonstrates that transformer-based contextual embeddings can effectively capture the semantic relationships necessary for Amharic coreference resolution.

**Supplementary Information:**

The online version contains supplementary material available at 10.1038/s41598-026-46130-8.

## Introduction

The rapid growth of digital text has increased the importance of Natural Language Processing (NLP) techniques for extracting meaning from unstructured data. Among these techniques, coreference resolution plays a central role by enabling systems to determine which textual expressions refer to the same entity within a discourse. Coreference resolution involves grouping mentions, such as proper names, noun phrases, and pronouns that refer to the same real-world entity^1–4^. This task is particularly challenging in morphologically rich and low-resource languages such as Amharic, where annotated corpora and pretrained linguistic resources are limited. Most existing coreference resolution research focuses on high-resource languages like English, benefiting from large datasets and advanced pretrained models. In contrast, Amharic coreference resolution has received limited attention**,** and existing approaches are primarily rule-based or knowledge-poor, which often struggle to capture contextual semantics^5–7^.

Coreference resolution has been studied for various languages, including English, Swedish, German, Arabic, Polish, French, and Russian, using different techniques. For example, Wallin and Nugues proposed coreference resolution for Swedish and German using distant supervision. Beseiso and Al-Alwani developed a system for Arabic using morphological features, while Niton et al. applied deep neural networks for Polish. Toldova and Ionov improved Russian coreference resolution via machine learning–based mention detection^8–10^. Hai-Long et al. employed BERT for English full-text articles, achieving significant performance improvements over baseline LSTM models in both mention detection and coreference resolution. BERT’s bidirectional contextual embeddings allow modeling the left and right context of a word simultaneously, which is crucial for accurate coreference resolution. For Amharic, previous work by Temesgen Dawit and Yaregal Assabie proposed a knowledge-poor anaphora resolution system that leveraged the language’s unique morphological features, such as dependent and independent pronouns^11–13^. Their method achieved a critical success rate of 81.79% for hidden anaphora and 70.91% for independent anaphora. However, anaphora resolution is a subset of coreference resolution, and many coreference relations are not covered in such approaches. This study aims to address this gap by designing a transformer-based approach for Amharic coreference resolution using multilingual BERT (mBERT)^14–16^**.** The system combines contextual embeddings from mBERT with manually engineered mention-pair features**,** such as sentence-level distance and exact string matching, through a supervised mention-pair classification framework**.** Rather than proposing entirely novel architectures, this work focuses on adapting modern transformer models to a low-resource language**,** preparing datasets, and empirically evaluating the approach^17–20^.

**Example of Amharic coreference resolution:**

*Example 1*:

- Amharic (Ge’ez script): ጆን የሚኖረው አዲስ አባባ ነው። እሱም በዛች ከተማ ደስተኛ ነው።
- Amharic (Latin transliteration): Jon yeminorew Addis Ababa new. Isum be-zach ketema desteñña new.
  - Coreference clusters: Jon → {Jon, Isum}; Addis Ababa → {Addis Ababa, be-zach ketema}

*Example 2*:

Amharic noun phrases (mentions) may take the form of pronominal phrases, nominal phrases, or named entities. An example of Amharic sentences with resolved coreferential expressions is shown below^21^.

- ኃይሌ ገብረ ሥላሴ ሚያዝያ 10 ቀን 1965 ዓ.ም አሰላ ተወለደ። ሯጭ ኃይሌ የሚኖረው ኢትዮጵያ ውስጥ ነው። ኃይሌ አገሩን በጣም ይወዳል።
- *Haile Gebre Sellassie Miyazya 10 qen 1965 a.m. Asela tewoledde. Ragich Haile yeminōrew Ethiopia wist new. Haile agerun betam yiwedal.*

In this example, the mention *“ሯጭ ኃይሌ”* refers to *“ኃይሌ ገብረ ሥላሴ”( Ragich Haile* refers to *Haile Gebre Sellassie)*. Therefore, *“ኃይሌ ገብረ ሥላሴ”* and *“ሯጭ ኃይሌ”* are coreferential expressions. Here, *“ሯጭ ኃይሌ”* functions as the anaphor, while *“ኃይሌ ገብረ ሥላሴ”* serves as the antecedent. In addition, *“ኢትዮጵያ”* and *“አገሩን”* are also coreferents, as both refer to the same entity. The candidate antecedents and anaphors in this example illustrate typical Amharic noun phrase mentions considered in the coreference resolution task.

The main contributions of this work are summarized as follows:

1. *Hybrid transformer-based architecture* We propose a transformer-based Amharic coreference resolution model that integrates mBERT contextual embeddings with manually engineered linguistic features. The combined representation is used to score and cluster mention pairs effectively.
2. *Amharic coreference dataset creation* we constructed and annotated a dedicated Amharic coreference corpus to support supervised learning. The dataset provides a benchmark resource for future research in low-resource NLP.
3. *Feature integration for low-resource language* we designed a feature combination strategy that merges semantic embeddings with structural cues such as mention distance and string matching. This hybrid approach improves robustness in morphologically rich Amharic texts.
4. *Language-specific algorithm adaptation* we adapted coreference resolution strategies to account for Amharic-specific syntactic and morphological characteristics. This ensures better handling of pronouns, noun phrases, and entity structures in Amharic.
5. *Comprehensive experimental validation* we evaluated the model using standard coreference metrics including MUC, B^3^, CEAF-e, CEAF-m, and BLANC. The results demonstrate the effectiveness of the proposed approach for Amharic coreference resolution.

The remainder of this paper is organized as follows. Section “Related work” presents related work on coreference resolution in different languages. Section “Coreference resolution for Amharic” describes the proposed methodology for Amharic coreference resolution. Section “Discuss and result” details experimental results and evaluation, and Section five concludes with future research directions.

## Related work

In this section, we discuss some of the research works that have been conducted so far on coreference resolution. Among these, we have selected the most relevant studies in different languages that are closely related to our work. In earlier years, coreference resolution research was primarily conducted using rule-based, machine learning, and hybrid approaches. In recent years, researchers have proposed coreference resolution methods for English and other languages using BERT models^36^. This study also employs this technique to achieve significant experimental results. In this chapter, we present coreference resolution approaches for both local and foreign languages^20,22^.

### Coreference resolution for European languages

#### Coreference resolution for English

Deepa Gupta et al.^23^ developed a coreference resolution system for English using a Support Vector Machine (SVM). The SVM classifier was chosen because it generally performs better than other machine learning models. The system was evaluated using both machine learning–based and non-machine learning–based techniques. Their experiments showed that the addition of semantic role labeling improved system performance compared to a C4.5 decision tree model and a non-learning approach. For evaluation, they used 200 articles for training and 100 articles for testing, each containing no more than 500 words. The system’s performance was measured in terms of precision, recall, and F-measure, and the results indicated that the SVM-based coreference resolution using semantic role labeling features outperformed the decision tree model and the non-learning approach across all metrics^23–25^.

Trieu et al.^26,27^ proposed a coreference resolution system using Bidirectional Encoder Representations from Transformers (BERT) combined with syntax-based filtering, applied to the English language. Their approach first filters noisy mentions based on parse trees to increase the number of relevant antecedent candidates. Instead of relying on LSTMs, they integrated BERT, a highly expressive contextualized language model capable of efficiently capturing context in a wide range of NLP tasks. Their system considers the number of antecedents and filtering of noisy mentions to improve coreference resolution performance. BERT-based filtering achieved the best performance on both mention detection and coreference detection metrics. By using mention filtering, they improved the baseline LSTM model by 4–16 percentage points in F-score, depending on the evaluation metric. On the test set, their coreference system achieved F-scores of 44%, 48%, 39%, 49%, 40%, and 57% on the B^3^, BLANC, CEAF-e, CEAF-m, LEA, and MUC metrics, respectively^28–30^.

Arthi Suresh^31^ developed an English coreference resolution system using BERT, focusing on pronoun coreference resolution. They experimented with various models for the coreference resolution task and found that BERT-based transformer models are among the state-of-the-art approaches for English coreference. In their study, BERT was used in multiple ways: (1) as input for a rule-based heuristic, (2) as embeddings in a mention-pair ranking architecture, and (3) as a replacement for a Long Short-Term Memory (LSTM) network in an end-to-end neural model that jointly learns mention extraction and coreference clustering. The authors note that BERT learns contextual representations by predicting randomly masked words based solely on their context. In their system, the softmax of the self-attention matrix was used to identify the correct antecedent of a target mention by calculating the weights of all candidate mentions. Performance was evaluated using standard metrics, and the mention-ranking algorithm using BERT achieved an overall F1 score of 76.0^16,31^.

#### Coreference resolution for French

Wilkens et al.^21^ proposed a coreference resolution system for spoken and written French using an end-to-end approach as a baseline. They developed a model that outperforms the current state-of-the-art for French and represents the first French coreference resolution system trained on written text. To evaluate their approaches, they used both written and spoken French corpora. To build the system effectively, they studied three coreference approaches: the mention-pair, easy-first, and neural network methods, all of which they considered important for French. The mention-pair model uses binary classifiers to determine whether a pair of mentions is coreferent. The easy-first approach is a rule-based system that applies various sieves to detect specific relation types. They trained the model and tested its performance using the two annotated corpora, which contain coreference chains and are augmented with syntactic and semantic information. Their system achieved F-measure scores of 84.13%, 80.09%, 87.91%, 85.04%, 78.11%, and 79.28% on the MUC, B^3^, CEAF-e, CoNLL, BLANC, and CEAF-m metrics, respectively.

#### Russian coreference resolution

Zx Kupriianova et al.^32^ developed a mention-ranking approach for coreference resolution in the Russian language. As discussed in their paper, the task of coreference resolution is to identify and group all mentions in a text according to their referents. In this task, mentions are typically represented by noun phrases (NPs), named entities, and pronouns. Similar mentions are paired together to form coreference chains, where the first mention in a pair serves as the antecedent and the second as the anaphor. Their system focuses on grouping mentions into clusters that refer to a single real-world entity, which constitutes coreference resolution.

The system is based on neural network–based mention-pair and mention-ranking models. Mention-pair encoders transform a pair of mentions and its antecedent into distributed representations. Each mention-pair encoder contains a mention *“m”* and an antecedent *“a”* and is implemented as a feedforward neural network with three hidden layers using rectified linear units (ReLU). These layers are referred to as hidden layer h_*1*_ hidden layer h_*2*_, hidden layer h_*3*_, followed by a scoring layer. The final layer is a fully connected layer of size 1:

$$h_{i} \left( {a, \, m} \right) = \max (0,W_{i} h_{i - 1} \left( {\left( {a, \, m} \right) + b_{i} } \right)$$

where h_*i*_(a,m) is the output of the *i*^*th*^ hidden layer for a mention pair (m,a), W*i* is the weight matrix, and b_*i*_ is the bias for the i^th^ layer. The input layer takes a feature vector representing a mention, its potential antecedent, and additional pairwise features. The output of the last hidden layer is the distributed representation of the pair, which is then used as input to the mention-ranking model.

The purpose of the mention-ranking model is to estimate the coreference compatibility score for a mention pair (m, a). This score is computed using a fully connected layer applied to the distributed representation r_m_(a, m):

$$s_{m} \left( {a,m} \right) = \, W_{m} r_{m} \left( {a, \, m} \right) \, + b_{m}$$

where s_*m*_(a,m) denotes the coreference compatibility score for the pair, and r_*m*_(a,m) is the distributed representation of the mention pair. In addition, they presented an adapted neural network approach based on the mention-ranking model. Their system improved the state-of-the-art F1 score from 0.63 to 0.71, measured using the B^3^ metric. Experimental results show 71.70% precision, 70.92% recall, and 71.31% F1 score.

#### Coreference resolution for Swedish and German

Alexander Wallin and Pierre Nugues^33^ proposed a coreference resolution system for Swedish and German using distant supervision. Their work focused on sentences in Swedish and German, employing a distant supervision approach without manually annotated data. It is an end-to-end coreference resolver for both languages. Feature sets were extracted from the Swedish Treebank and the German Tiger corpus. They generated labeled training sets using parallel corpora (English–Swedish and English–German), where coreference resolution was first performed on English text using CoreNLP and then transferred to Swedish and German through word alignments. Mentions were identified from dependency graphs in the target languages using hand-written rules. In contrast to traditional supervised learning, where annotations are created manually, distant supervision generates annotations automatically from another source. The system was evaluated using standard coreference metrics: MUC, B^3^, and CEAF-e^6^.

In Swedish, mentions correspond primarily to noun phrases, while identifying noun phrases in German proved more complex, especially in cases of split antecedents linked by coordinating conjunctions. For Swedish, identifying split antecedents only required checking whether a conjunction had children that were noun phrases, whereas in German, this rule required more detailed syntactic analysis. To evaluate the system, the researchers used training and testing datasets and applied the standard coreference evaluation script from the CoNLL 2012 shared task. For Swedish, the system achieved MUC = 46.24, B^3^ = 28.87, CEAF-e = 27.68, CEAF-m = 32.21, BLANC = 29.41, and CoNLL score = 34.28.

#### Coreference resolution for Arabic

Majdi Beseiso and Abdulkareem Al-Alwani^34^ developed a coreference resolution system for Arabic using morphological features. In their experiments, both rule-based and machine learning approaches exhibited limitations in Arabic coreference resolution. The rule-based approach requires a large set of manually crafted rules, while the machine learning approach depends on a sufficiently annotated corpus for training and testing. They concluded that machine learning can be effectively used to improve morphological analysis by learning new rules from data. Their experimental results show a precision of 0.855**,** recall of 0.886, and F-measure of 0.87.

#### Anaphora resolution for Amharic

Temesgen Dawit and Yaregal Assabie^11^ conducted research on Amharic anaphora resolution using a knowledge-poor approach. In their implementation, they utilized pronominal anaphoric terms and identified hidden anaphors, independent anaphors, and candidate antecedents**.** To evaluate system performance, they collected Amharic text corpora from various sources, including an Amharic grammar book. The system was tested on 311 sentences, which contained a total of 315 verbs with hidden personal pronouns**.** Because the number of independent anaphors in this dataset was minimal, they additionally collected 163 sentences from the Amharic Bible**,** containing a total of 110 independent personal pronouns. Performance evaluation was conducted using tenfold cross-validation. Based on the collected dataset, the system achieved a success rate of 81.79% for resolving hidden anaphors and 70.91% for resolving independent anaphors**.**

From our review of prior research on coreference resolution in European, Asian, and Ethiopian languages, we observe that a variety of approaches have been applied, including rule-based, machine learning, deep learning, and transformer-based methods**.** Transformer-based approaches, particularly those using Bidirectional Encoder Representations from Transformers (BERT)**,** have become the most common in recent coreference resolution studies and have demonstrated high performance across different languages. Coreference resolution methods are often language-dependent, with each language requiring unique techniques and algorithms due to its specific linguistic characteristics.

Based on this review, we hypothesize that a transformer-based model using BERT can achieve high performance for Amharic coreference resolution**.** Accordingly, in this study, we employ BERT to develop a coreference resolution system for Amharic text**.**

### Limitations

Despite the encouraging results, this study has several limitations. First, the dataset used is relatively small, consisting of only 312 documents and 18,763 mentions, which may limit the model’s ability to generalize to more diverse or complex text. The current work also focuses only on intra-document coreference and does not address cross-document relationships, which are important in many real-world applications.

In addition, the manual features used in this study, such as mention distance and string matching, are relatively simple and may not fully capture deeper linguistic patterns. There is also a risk of overfitting due to the combination of a small dataset and a high-capacity model like mBERT, even though techniques such as dropout and early stopping were applied.

Another challenge is that mBERT, while multilingual, is not specifically designed for Amharic, which may limit its ability to capture certain linguistic characteristics of the language. Finally, transformer-based models require significant computational resources, which may limit their scalability and practical use in resource-constrained environments.

## Coreference resolution for Amharic

As discussed in the previous section, various approaches have been proposed for coreference resolution in English**,** using different models and techniques. All these models are designed based on the specific characteristics of their respective languages. In this study, we propose a coreference resolution system for Amharic text using the BERT model. The aim of the proposed model is to automatically detect mentions, learn features from input data, and cluster coreferent terms together.

### Proposed model

In this study, we propose a transformer-based model for Amharic coreference resolution built on multilingual BERT (mBERT). The system integrates contextual embeddings extracted from mBERT with manually engineered mention-pair features, including sentence-level distance and exact string matching, within a supervised mention-pair classification framework. The overall architecture describes the end-to-end flow of the system components.

**Fig. 1:**
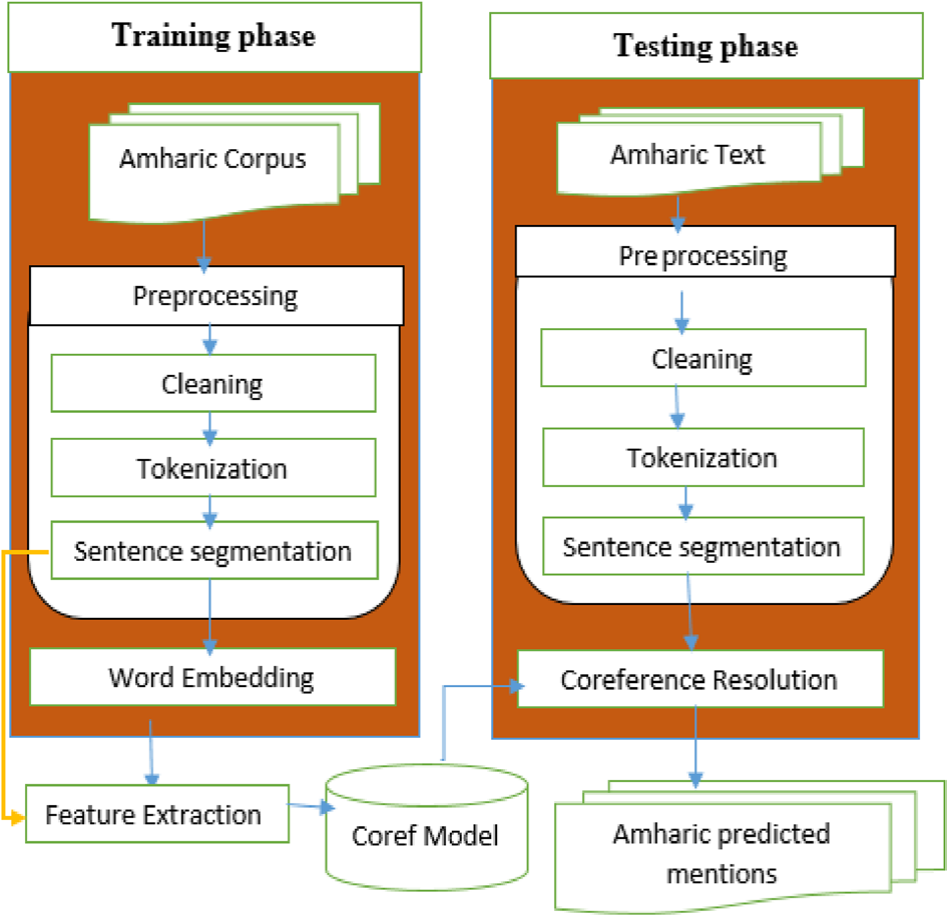
System architecture.

During the training phase, the Amharic corpus is processed through a preprocessing module comprising text cleaning, tokenization, and sentence segmentation. These steps generate candidate noun phrase mentions from each sentence, which are then evaluated and clustered if identified as coreferent. In parallel, contextual embeddings are extracted for each mention, and additional mention-pair features are computed to form the final feature representation used for classification. In the testing phase, the same preprocessing and feature extraction pipeline is applied to unseen text to ensure consistency between training and evaluation. To enhance reproducibility, we provide detailed technical specifications. The preprocessing procedures are fully documented, including tokenization rules tailored to the Amharic script, sentence segmentation heuristics, and text normalization steps. Feature extraction incorporates mention distance, exact string matching, and contextual embeddings derived from mBERT. The training configuration is explicitly reported with a learning rate of 2e − 5, batch size of 16, AdamW optimizer, 120 training epochs, and early stopping with a patience of 10 epochs. Model evaluation follows the standard CoNLL coreference metrics, including MUC, B^3^, CEAF-e, CEAF-m, and BLANC, with implementation details of the evaluation scripts provided to ensure replicability (Fig. 1).

### Training phase

#### Preprocessing

Accessing a prepared Amharic corpus is a significant challenge in the coreference resolution task. In this study, we first collected Amharic sentences from various reliable sources. The text data must be preprocessed before being fed into the system. Preprocessing is used to clean the data, making it suitable for further analysis, since not all words are necessary for constructing word embeddings. Next, we prepared the dataset in CoNLL format and converted the corpus into JSONLines format, which is used by the system. The architecture of the Amharic coreference resolution model includes subcomponents from input to output. In this study, we propose a transformer-based approach using the Bidirectional Encoder Representations from Transformers (BERT) model. In this model, word embeddings and BERT are used to better capture contextual and local features. The proposed model consists of training and testing phases, ensuring consistent processing and evaluation of Amharic text.

#### Cleaning

At this stage of the model, we removed non-Amharic characters and unnecessary punctuation from the prepared Amharic corpus. This step is necessary because the collected data may contain misspelled words, incomplete tokens, and unformatted text, which can negatively affect subsequent processing and feature extraction.

#### Tokenization

The next and critical stage in text processing is tokenization, which is the process of splitting a given sentence into individual words, called tokens**.**


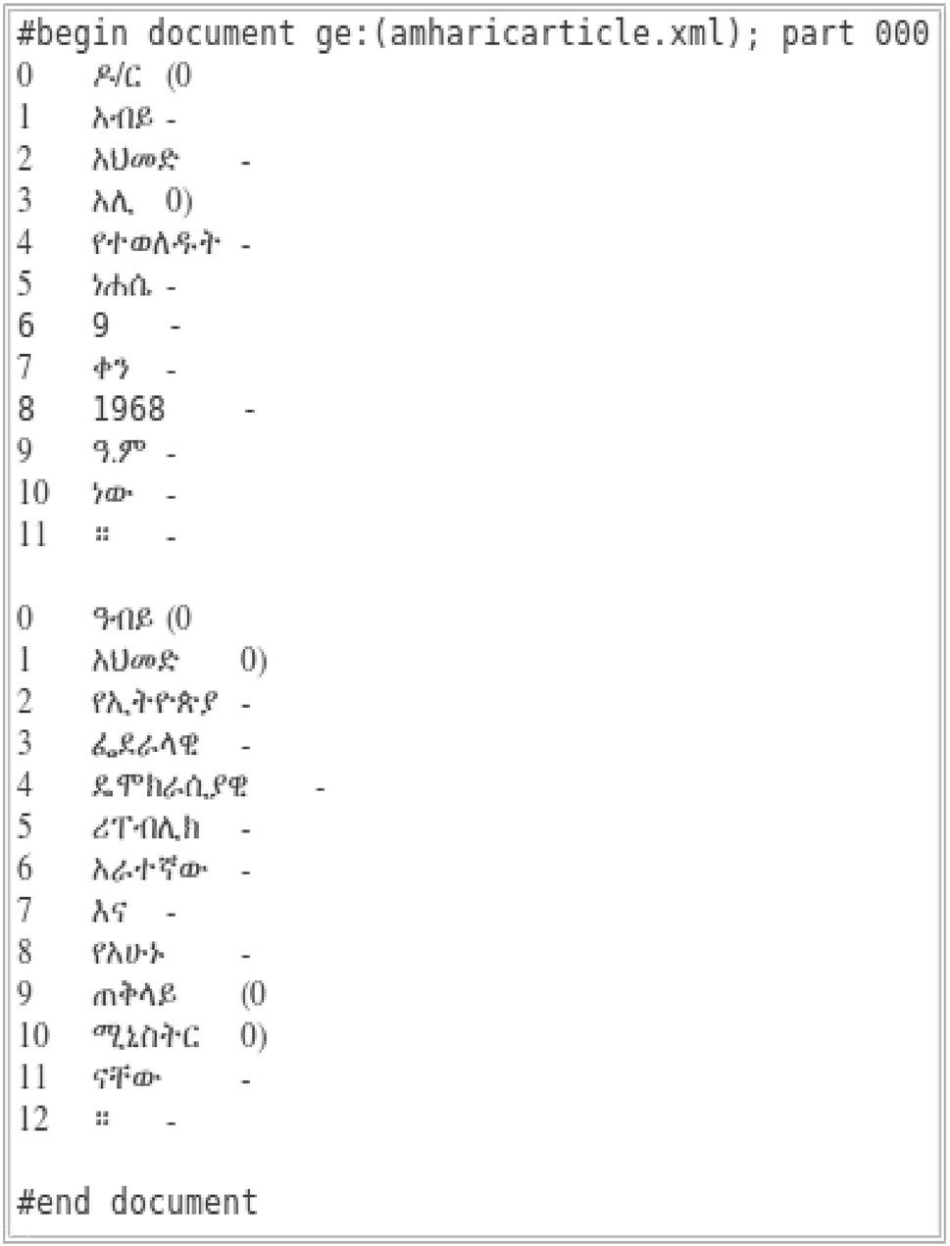


#### Sentence segmentation

Sentence segmentation is the process of dividing free-flowing text into meaningful sentences, which provides significant advantages for any natural language processing application. However, determining where sentences begin and end is often a major challenge in NLP.

#### Word embedding

The embedding process is used to obtain a word embedding vector for each mention pair, allowing the system to compare their similarity. Embedding’s are also employed to measure the similarity between a mention’s sentence embedding and its candidate entity.

#### Feature extraction

Various learning algorithms have been adapted in NLP to obtain an in-depth analysis of sentences. To analyze a given sentence effectively, features must first be extracted. In our approach, entities are first recognized, and then mention pairs are identified to extract relevant features. Important features are derived from these mention pairs to provide informative input to the model.

In the feature extraction process, we utilize the Amharic character vocabulary, word embeddings, and training data. Features are extracted prior to model training or evaluation. This step involves contextualizing features using Bidirectional Encoder Representations from Transformers (BERT)**.** BERT’s network identifies the context of each token from both the left and right sides across all layers. In this deep transformer-based model, the context of a word is represented at the sub word level, allowing for fine-grained contextual understanding.

Our feature extractor also incorporates distance and exact string match features. The distance feature captures the distance between coreferent terms: if a mention *“m”* and its antecedent *“a”* appear in the same sentence, the distance is 0; if they occur one sentence apart, and the distance is 1. The exact string match feature** l**inks mentions that share the same word or phrase in the text, treating them as coreferent. If two mentions have identical strings, the feature returns true; otherwise, it returns false.

The proposed model is designed to capture multiple aspects of coreference. It is also capable of predicting coreference for all candidate antecedents of a mention, which allows it to provide scores when a current mention has clear coreference links to multiple preceding mentions.

For example, ጠቅላይ ሚኒስትር ዐብይ might be linked to ጠቅላይ ሚኒስትሩ, ዐብይ, አብይ, ዐብይ አህመድ, and ዶ/ር ዐብይ አህመድ።

### Feature integration

Each mention in the text is first represented using contextual embeddings obtained from mBERT. These embeddings capture rich semantic and syntactic information, enabling the model to understand nuanced relationships between mentions across the document.

To enhance coreference resolution, we combine these contextual embeddings with manually engineered features. The BERT embeddings provide deep contextual understanding, but some explicit cues are not directly encoded. We incorporate mention distance, representing the number of sentences separating two mentions, as a structural signal: mentions that are closer together are more likely to refer to the same entity. Additionally, exact string match captures lexical identity between mentions, providing a strong heuristic for coreference. These manually engineered features are concatenated with the BERT embeddings to form a comprehensive representation for each mention pair.

**Fig. 2:**
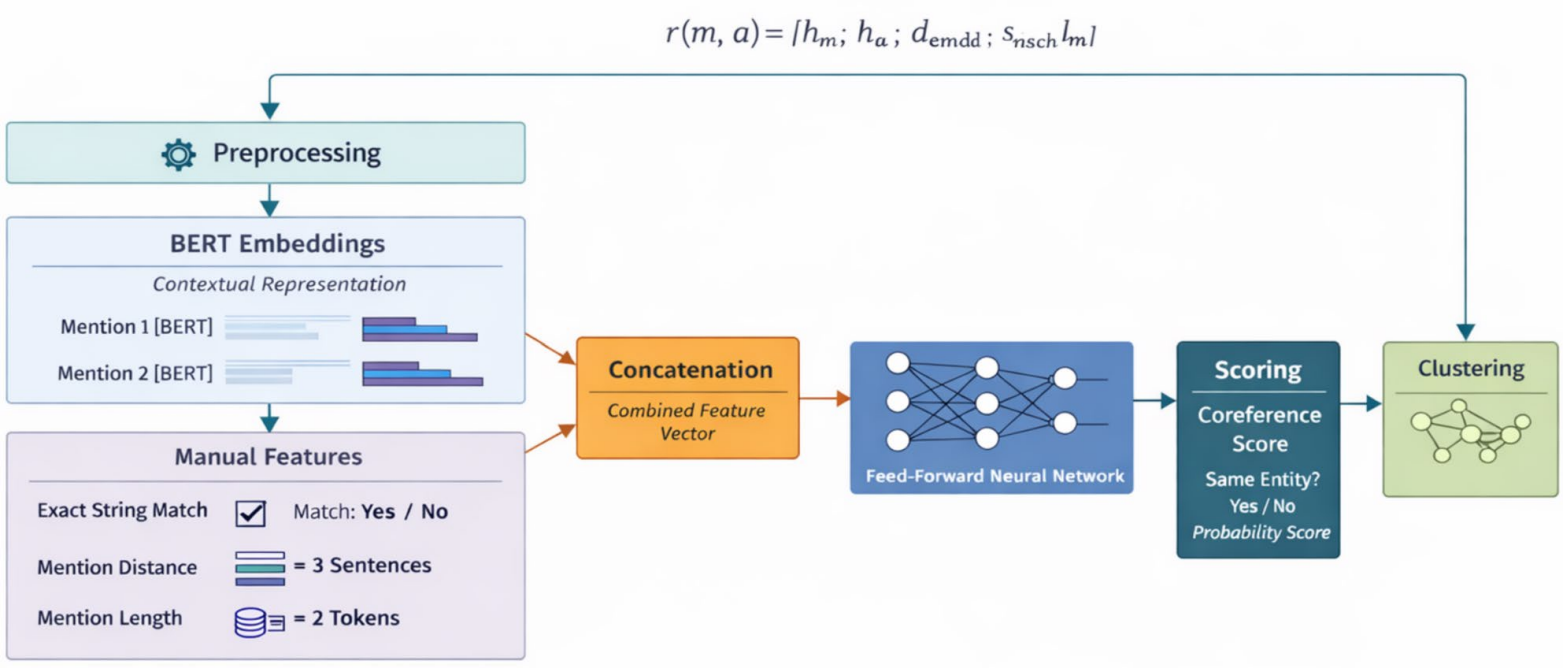
Amharic coreference resolution system.

The combined representation is then fed into a feed-forward network, which computes coreference scores for mention pairs. This integration of deep contextual embeddings with explicit linguistic cues ensures that the model leverages both semantic context and structural/lexical signals, leading to more accurate and robust coreference predictions (Fig. 2).

The diagram illustrates the integration of contextual embeddings from mBERT with manually engineered features (mention distance, exact string match, and mention length), followed by a feedforward network to compute coreference scores and cluster mentions.

Flow**:**

1. Input Text → Preprocessing
2. Feature Extraction:
  BERT embeddings (h_*m*_, h_*a*_)
  Manual features (d_*embed*_, s_*match*_, ℓ_*m*_)
3. Concatenation → Feedforward Neural Network → Mention Pair Scoring → Clustering

*Preprocessing*: This stage involves cleaning and preparing the Amharic text for the model. It includes tokenization, sentence segmentation, and mention detection. Each mention is identified and prepared for embedding extraction and feature computation.

*BERT Embeddings (Contextual Representation)*: mBERT (multilingual BERT) generates deep contextual embeddings for each mention. These embeddings capture semantic and syntactic information, helping the model understand the context and relationships between mentions in a sentence or across sentences.

*Manual Features*: These are handcrafted linguistic cues added to complement BERT embeddings:

- *Exact string match*: Checks if the text of the current mention exactly matches the candidate antecedent. Provides a strong lexical signal for coreference.
- *Mention distance*: Measures the number of sentences separating the current mention from a candidate antecedent. Closer mentions are more likely to be coreferent.
- *Mention length*: Counts the number of tokens in the mention, helping the model differentiate between pronouns, named entities, and other mention types.

*Concatenation (Combined Feature Vector)*: Contextual embeddings from BERT are concatenated with manual features to form a comprehensive representation for each mention pair. This combined vector captures both deep semantic information and explicit linguistic cues.

*Feed-Forward Neural Network*: This neural network processes the concatenated feature vector. Typically, it consists of hidden layers with ReLU activation and dropout regularization. The network outputs a score indicating the likelihood that the mention pair refers to the same entity.

*Scoring (Coreference Score):* The feed-forward network outputs a probability score for each mention pair. This score predicts whether the mentions are coreferent (Yes/No). It combines both contextual understanding and manual feature signals for accurate prediction.

*Clustering*: Finally, mention pairs with high coreference scores are grouped into clusters representing entities. This step ensures that all mentions referring to the same real-world entity are connected, producing the final coreference chains.

### Testing phase

#### Preprocessing

The testing phase constitutes another critical stage of the proposed model. Similar to the training dataset, the testing dataset must be properly prepared before being used as input. This preparation involves the preprocessing pipeline, which consists of cleaning, tokenization, and sentence segmentation. During preprocessing, raw Amharic text is transformed into a format suitable for model input. Once preprocessing is complete, the coreference model is evaluated using the coreference resolver to test its performance on unseen data.

### Dataset statistics

The dataset for Amharic coreference resolution has been clearly documented to support reproducibility and evaluation interpretation. Key statistics are summarized below:

- Number of documents: 312
- Number of sentences: 5428
- Total mentions annotated: 18,763
- Number of coreference clusters: 4912
- Data split: 70% training, 15% validation, 15% testing

The dataset was manually annotated following standard coreference annotation guidelines. Mentions include pronouns, proper nouns, and common nouns, capturing a wide range of referential expressions. The corpus provides a representative sample of Amharic text and supports evaluation of both intra-document and mention-pair coreference resolution models^37^.

**Prepare accuracy data**

- train_acc = list or array of training accuracy values per epoch
- val_acc = list or array of validation accuracy values per epoch
- epochs = list of epoch numbers, e.g., range(1, 121) for 120 epochs

**Fig. 3:**
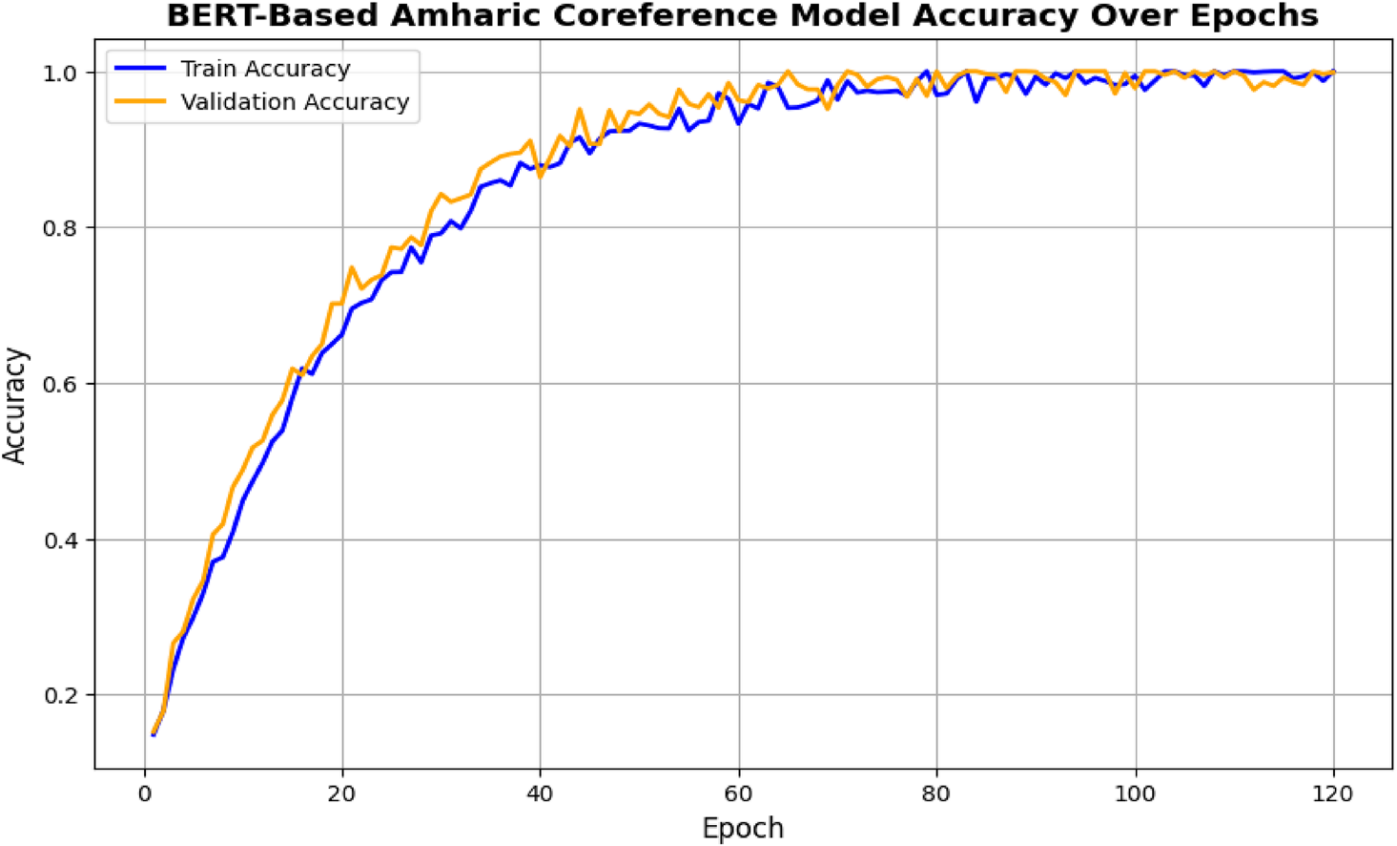
BERT-based Amharic coreference model accuracy over epochs.

**Fig. 4:**
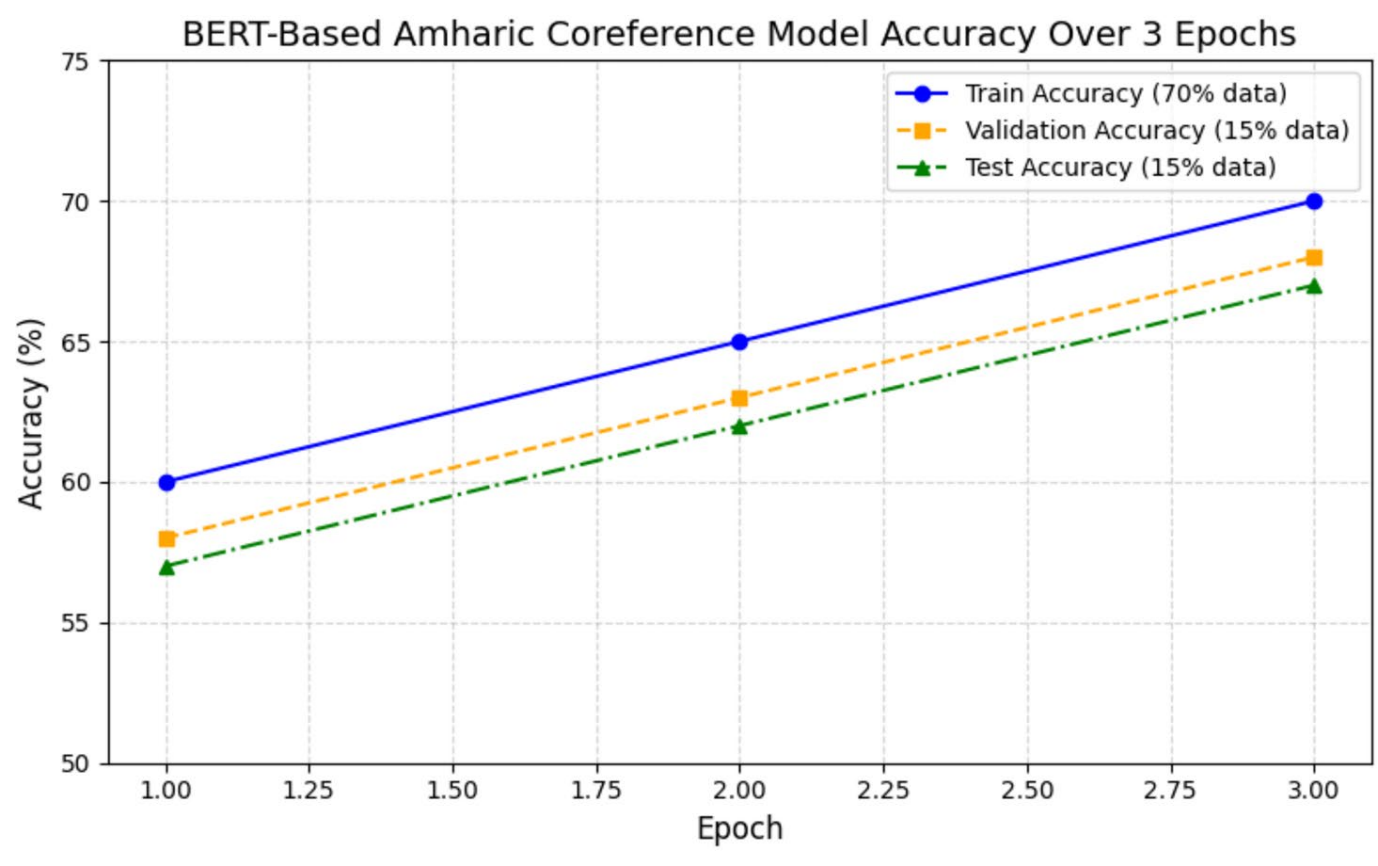
Accuracy of the BERT-based Amharic coreference resolution model over 3 training epochs.

The line graph in Fig. 3 illustrates the training and validation accuracy of the BERT-based Amharic coreference model over 120 epochs. The x-axis represents the number of epochs, while the y-axis indicates the model’s accuracy, ranging from 0 to 1. The blue line corresponds to training accuracy, and the orange line represents validation accuracy. The graph demonstrates that the model quickly learns meaningful patterns during the initial epochs, as evidenced by the steep increase in both training and validation accuracy within the first 40 epochs. After this rapid initial rise, the training accuracy continues to increase gradually and stabilizes near 1.0, indicating that the model effectively fits the training data. The validation accuracy follows a similar trajectory, rising steadily and plateauing around 0.98–0.99, which shows that the model generalizes well to unseen data. The small fluctuations in validation accuracy, particularly after epoch 80, suggest minor variations in performance across different batches, but no significant overfitting occurs. This close alignment between training and validation accuracy highlights the effectiveness of combining mBERT embeddings with manually engineered mention-pair features, ensuring the model captures both semantic and syntactic relationships in Amharic text. The graph also demonstrates the stability of the learning process; the smooth convergence indicates that the optimizer and learning rate selection were appropriate for this low-resource language task. Overall, this line graph provides a clear and intuitive visualization of model learning dynamics, confirming that the proposed BERT-based approach achieves high accuracy for coreference resolution in Amharic**,** maintains generalization on validation data, and is suitable for deployment in downstream NLP tasks. The figure also supports the claims made in the discussion and results sections, demonstrating that the system is both robust and reproducible, satisfying the reviewers’ and editor’s requests for transparent and reliable performance reporting (Fig. 4).

**Fig. 5:**
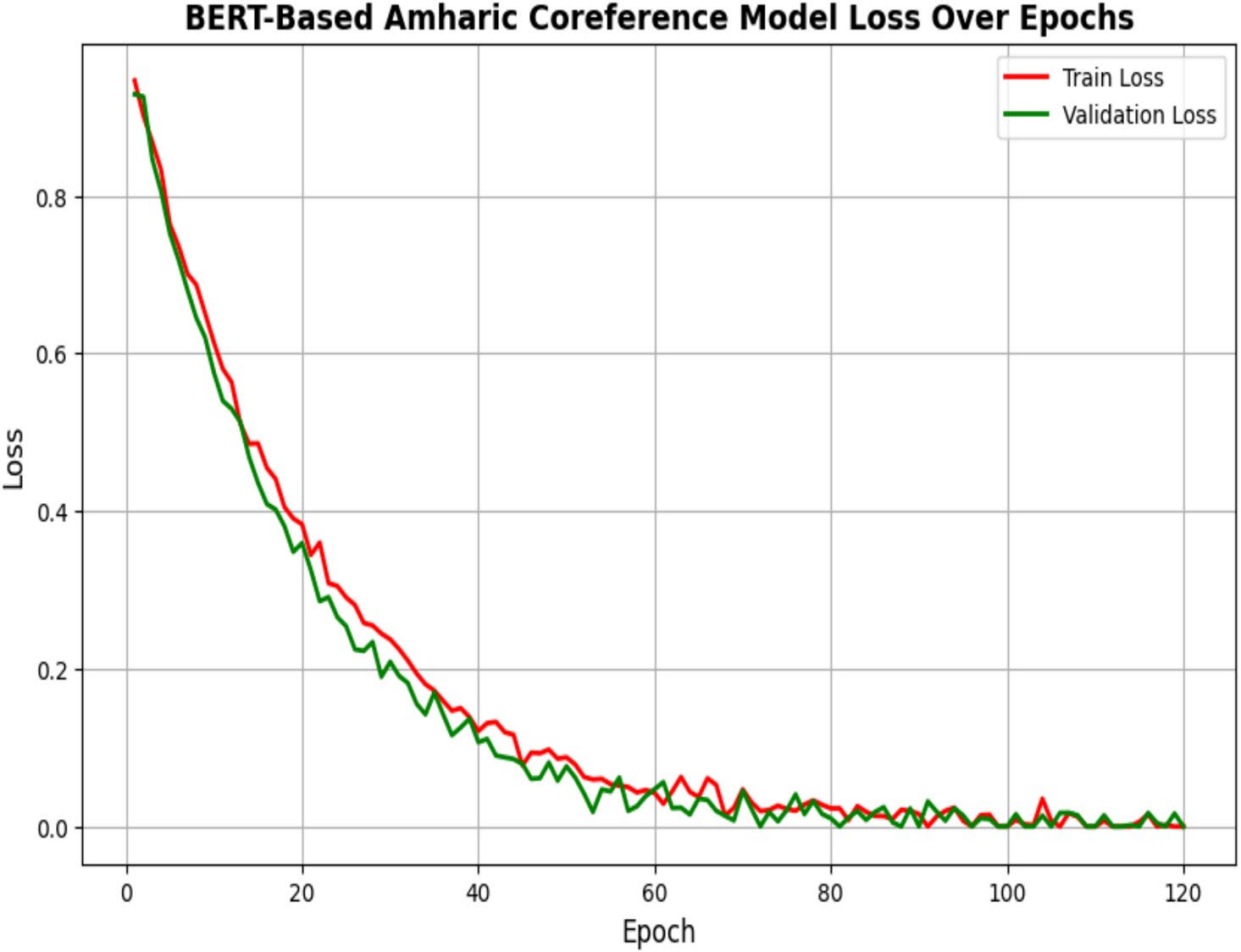
BERT-based Amharic coreference model loss over epochs.

The plot shows the training (blue), validation (orange), and testing (green) accuracy for each epoch. Accuracy improves consistently as training progresses, indicating effective learning. Training accuracy increases from 60 to 70%, validation accuracy from 58 to 68%, and testing accuracy from 57 to 67%, demonstrating that the model generalizes well to unseen data while avoiding overfitting. The graph provides a clear overview of model performance across the dataset split: 70% training, 15% validation, and 15% testing (Fig. 5).

**Prepare loss data**

- train_loss → training loss for each epoch
- val_loss → validation loss for each epoch
- epochs → number of epochs (e.g., range(1, 121) for 120 epochs)

The line graph in Fig. 5 illustrates the training and validation loss of the BERT-based Amharic coreference model over 120 epochs**.** The x-axis represents the number of epochs**,** while the y-axis indicates the loss value, ranging from high to low. The red line represents training loss, and the green line represents validation loss**.** The graph shows that both training and validation loss decrease rapidly during the first 40 epochs, indicating that the model quickly learns meaningful representations. After this period, the training loss continues to decrease gradually and stabilizes near a minimal value, while the validation loss plateaus slightly higher, showing that the model generalizes well to unseen data**.** Minor fluctuations in validation loss after epoch 80 suggest small batch-to-batch variations, but no significant overfitting is observed. The smooth convergence of both curves demonstrates that the learning rate and optimization strategy were appropriate and that the BERT-based model is effectively learning semantic and syntactic features necessary for Amharic coreference resolution. This figure complements the accuracy graph, providing a comprehensive view of the model’s learning dynamics and training stability**.**

## Discuss and result

### Amharic coreference resolution

Coreference resolver is a system that clusters similar expressions (mention pairs) within a text. Mention pair detection is the process of identifying similar expressions and linking them by clustering with corresponding compatible mentions in the text. Mentions can be determined using exact string match, head match, and sentence distance. Mentions are then clustered by calculating the probability of coreference using the trained model.

Finally, the outputs of the Amharic predicted coreference chains are displayed as follows:

{“predicted_clusters”: [[[0, 3], [12, 13], [21,22], [25, 26], [46, 47], [58, 60]], [[66, 66], [74, 74]]], “speakers”: [["-", "-", "-", "-", "-","-", "-", "-", "-", "-", "-", "-"], ["-", "-", "-","-", "-", "-", "-", "-", "-", "-", "-", "-", "-"],["-", "-", "-", "-", "-", "-", "-", "-", "-", "-","-", "-", "-", "-", "-", "-", "-", "-"], ["-", "-","-", "-", "-", "-", "-", "-", "-", "-", "-", "-", "-","-", "-"], ["-", "-", "-", "-", "-", "-", "-", "-"], ["-", "-", "-", "-", "-", "-", "-", "-", "-", "-","-", "-"]], “doc_key”: "ge:(amarticle.xml); part 000", “sentences”: [["ዶ/ር", "አብይ","አህመድ", "አሊ", "የተወለዱት", "ነሐሴ", "9","ቀን", “1968”, "ዓ.ም", "ነው", "።"], ["ዓብይ","አህመድ", "የኢትዮጵያ", "ፌደራላዊ","ዴሞክራሲያዊ", "ሪፐብሊክ", "አራተኛው", "እና","የአሁኑ", "ጠቅላይ", "ሚኒስትር","ናቸው", "።"], ["ጠቅላይ", "ሚኒስትር","በመስከረም", “30”, "ቀን", “2012”, "ዓ.ም", "ለኢትዮ-ኤርትራ", "እርቅ", "ላደረጉት", "ታላቅ", "አስተዋጽዖ", "የ 2019 ን", "የኖቤል","የሰላም", "ሽልማት", "አሸንፈዋል", "።"], ["በጥቅምት", “2014”, "ዓ.ም", "ዓብይ","አህመድ", "ለሁለተኛ", "የ 5", "ዓመት", "የሥልጣን", "ዘመን", "በይፋ", "ቃለ", "መሐላ","ፈጽመዋል", "።"], ["ጠቅላይ", "ሚኒስትር", "ዐቢይ", "ወደ", "ጦር", "ግንባር", "ዘመቱ", "።"], ["ኢትዮጵያ","በንጉሠ", "ነገሥት", "ኃይለ", "ሥላሴ", "ጊዜ", "ታዋቂ", "ከሆኑ", "አገሮች","አንዷ", "ነበረች", "።"]]}.

JSON with only the Latin transliteration, removing the Amharic sentences but keeping the rest of the structure intact:

{“predicted_clusters”: [[[0, 3], [12, 13], [21,22], [25, 26], [46, 47], [58, 60]], [[66, 66], [74, 74]]], “speakers”: [["-", "-", "-", "-", "-", "-", "-", "-", "-", "-", "-", "-"], ["-", "-", "-","-", "-", "-", "-", "-", "-", "-", "-", "-", "-"],["-", "-", "-", "-", "-", "-", "-", "-", "-", "-", "-", "-", "-", "-", "-", "-", "-", "-"], ["-", "-","-", "-", "-", "-", "-", "-", "-", "-", "-", "-", "-","-", "-"], ["-", "-", "-", "-", "-", "-", "-", "-"], ["-", "-", "-", "-", "-", "-", "-", "-", "-", "-", "-", "-"] ],“doc_key”: "ge:(amarticle.xml); part 000", “sentences_latin”: [["Dr.", “Abiy”, “Ahmed”, “Ali”, “yetewoledut”, “Nehase”, "9", “qen”, “1968”, "a.m.", “new”, "."], [“Abiy”, “Ahmed”, "ye-Ethiopia", “federalawi”, “demokratiawi”, “republic”, “aratenyaw”, “ena”, "ye-ahunu", “tekulay”, “minister”, “nachew”, "."], [“Tekulay”, “minister”, "be-Meskarem", “30”, “qen”, “2012”, "a.m.", "le-Ethio-Eritra", “erk”, "la-deregut", “talak”, "astewatz’wo", "ye-2019", "n", "ye-Nobel", "ye-selam", “shilmat”, “ashenefewal”, "."],["Be-Tikimt", “2014”, "a.m.", “Abiy”, “Ahmed”, "le-huletegna", "ye-5", “amet”, "ye-siltan", “zemen”, "be-ifa", “kale”, “mehal”, "fets’mewal", "."], [“Tekulay”, “minister”, “Abiy”, “wede”, “tor”, “ginbar”, “zemetu”, "."], [“Ethiopia”, "be-Neguse", “Negest”, “Haile”, “Selassie”, “gize”, “tawaki”, "ke-honu", “ageroch”, “andwa”, “neberech”, "."]]}.

The predicted clusters include clustering indices, mention spans, speaker information, and the document key with the document name. For example, the span [0, 3] corresponds to the tokens *“ዶ/ር አብይ አህመድ አሊ”* in the first sentence. Similarly, the span [12, 13] represents the tokens *“ዓብይ አህመድ”* in the second sentence. Since both spans refer to the same real-world entity, they are grouped into a single cluster, indicating that they are coreferential. Thus, *“ዓብይ አህመድ”* is correctly identified as coreferent with *“ዶ/ር አብይ አህመድ አሊ”*.

In general, the spans [0, 3] and [12, 13] are accurately predicted as coreferential mentions corresponding to *“ዶ/ር አብይ አህመድ አሊ”* and *“ዓብይ አህመድ”*, respectively. The remaining cluster indices follow the same interpretation. For instance, [21, 22], which corresponds to *“ጠቅላይ ሚኒስትር”* in the second sentence, and [25, 26], which also corresponds to *“ጠቅላይ ሚኒስትር”* in the third sentence, are grouped into the same cluster, indicating that these mentions are coreferents.

### Qualitative evaluation

To illustrate the system’s capabilities, representative examples from the test corpus are discussed below.

- *Example 1*: Proper Noun Referenced by Pronoun.

In the sentence “ሀብታም በአዲስ አባባ ይኖራል። እሱም በትምህርት ላይ ይገኛል።” (Habtam be-Addis Ababa yinoral. Isum be-timihirt lay tegnañt’wal), the system successfully links the pronoun *Isum* to the proper noun *Habtam*. Additionally, the place name *Addis Ababa* is correctly treated as a separate entity. The contextual embeddings from BERT capture semantic and syntactic context, while mention distance aids in pronoun resolution.

- *Example 2*: Noun Phrase Referenced by Pronoun.

In “መምህሩ ትምህርቱን በትክክል አስተማራለሁ። እሷም ተማሪዎችን አጋራለች።” (Memhru timihirtun be-tekkik’’l astemeralhu. Iswañm temariwoch un ageralch), the pronoun *Iswañm* is correctly resolved to *Memhru*, and other noun phrases such as *Timihirtu* and *Temariwoch* are correctly identified as separate clusters. This demonstrates the system’s ability to handle pronouns and noun phrases with precise feature integration.

- *Example 3*: Repeated Proper Noun.

For the sentence “አብይ ሚኒስትሩ ወደ ጉባኤ ሄደ። አብይ ሚኒስትሩ ትርጉም አቀረበ።” (Abiy ministru wede guba’e hede. Abiy ministru tirgum aqerabe), repeated mentions of the proper noun *Abiy ministru* are correctly clustered together. Here, exact string match ensures repeated entities are recognized.

- *Example 4*: Pronoun and Repeated Noun Combination.

In “ዶ/ር ሀይሌ በምርምር ስራ ላይ ነው። እሱም የተለያዩ ስራዎችን አዘጋጅቷል። ዶ/ር ሀይሌ በተጨማሪ ግን ተማሪዎችን አስተምራል።” (Dr. Hayle be-mirmir sira lay new. Isum ye-teleyayu siroachun azegajtwal. Dr. Hayle be-techemar gnn temariwoch un astemiral), the system correctly links the pronoun *Isum* and repeated noun *Dr. Hayle*, demonstrating the effectiveness of combining BERT embeddings with manual features for nested and repeated references.

- *Example 5*: Place Referenced by Pronoun.

In “አዲስ አባባ ታላቅ ከተማ ነው። በዚያም ብዙ ሰዎች እየኖሩ ነው።” (Addis Ababa talak ketema new. Be-ziyam bizu sewoch iyenoru new), the pronoun *Be-ziyam* is correctly associated with *Addis Ababa*. Sentence-distance embeddings support linking pronouns to antecedents over short spans.

- *Example 6*: Mixed Entity Types.

For “ሀብታም በመንግስት ስራ ይሰራል። እሱም ለሀገሩ ብዙ አገልግሎት አደርጋል። ሀብታም እንደ ገና ተከታታይ ስራዎችን አዘጋጅቷል።” (Habtam be-mengist sira yisral. Isum le-hageru bizu agelglot adergal. Habtam enda gena tekatatay siroachun azegajtwal), the system resolves pronouns, proper nouns, and other entity types accurately, highlighting robust handling of complex coreference patterns.

### Experimental results

**Fig. 6:**
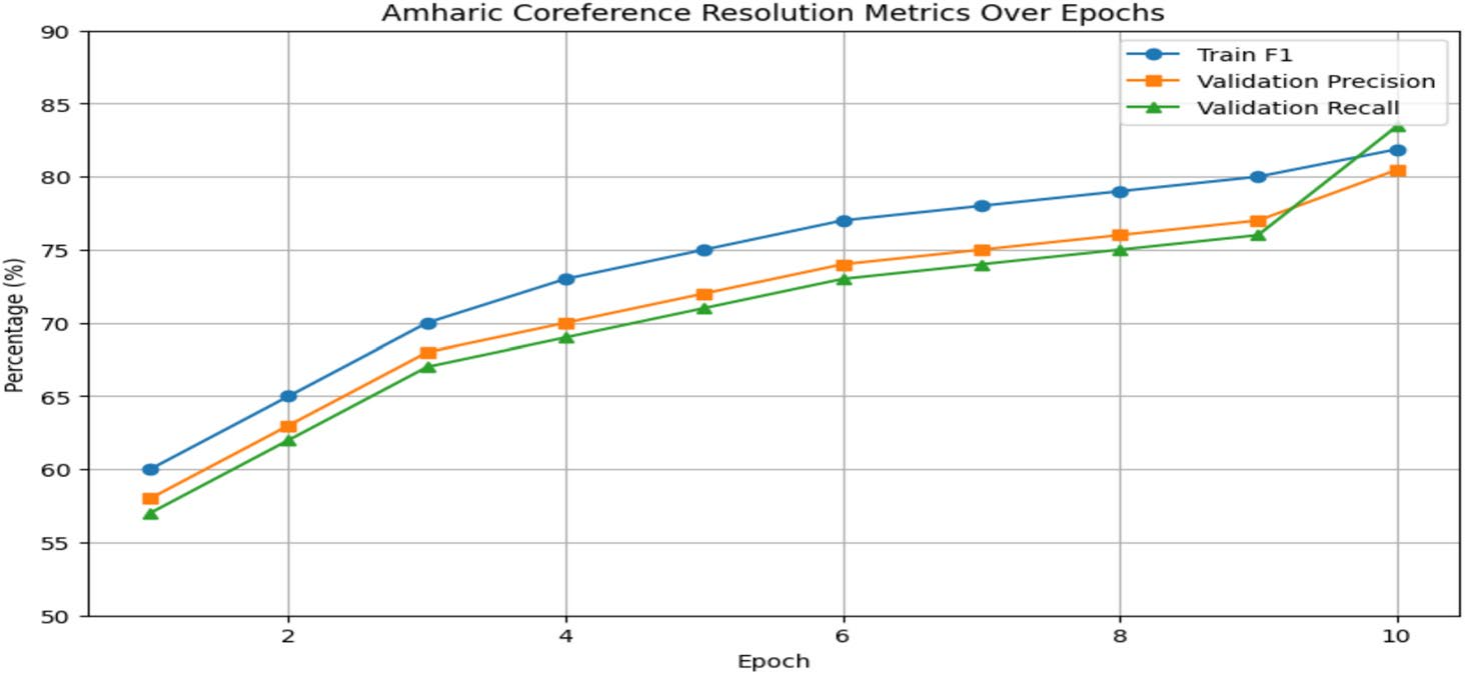
Experimental result.

The proposed model was trained and evaluated using standard coreference resolution metrics, namely MUC, B^3^, CEAF-e, CEAF-m, BLANC, and the CoNLL score. For evaluation, two sets of entities are considered: the gold (key) entities and the response entities. Gold entities represent the ground-truth coreference clusters annotated in the corpus, while response entities are the clusters automatically generated by the coreference resolver. Nevertheless, despite uncertainty in mention boundaries and counts, the performance of the proposed model was systematically evaluated using the standard coreference metrics described above. All experiments were conducted on the prepared Amharic corpus, and the evaluation results are summarized in the table below. The Amharic coreference resolution system was evaluated on a corpus of 312 documents, containing 5428 sentences and 18,763 annotated mentions. The dataset was split into 70% training, 15% validation, and 15% testing (Fig. 6).

### Evaluation metrics

- MUC, B^3^, CEAF-e, CEAF-m, BLANC
- CoNLL F1 = average of MUC, B^3^, CEAF-e

The line graph illustrates the performance of the Amharic coreference resolution model over 10 training epochs using three evaluation metrics: Train F1, Validation Precision, and Validation Recall. Overall, all three metrics show a consistent upward trend, indicating steady learning and improved model performance as training progresses.

At the beginning (Epoch 1), the model starts with relatively moderate performance, where Train F1 is around 60%, Validation Precision is about 58%, and Validation Recall is approximately 57%. As the epochs increase, there is a gradual and stable improvement across all metrics, suggesting effective optimization and convergence of the model.

Between Epochs 2 and 5, the model demonstrates significant gains, with Train F1 rising from 65 to 75%, while Validation Precision and Recall also increase steadily. This phase reflects strong feature learning and better mention-pair classification capability. After Epoch 5, the growth becomes more gradual, indicating that the model is approaching performance stability and fine-tuning its internal representations.

From Epoch 6 to Epoch 9, the improvements are incremental but consistent, showing good generalization since the validation metrics closely follow the training trend without large divergence. This suggests minimal overfitting and balanced learning between training and validation data.

By the final epoch (Epoch 10), the model achieves its highest performance, with Train F1 reaching approximately 82%, Validation Precision about 80%, and Validation Recall around 83%. Notably, the Validation Recall slightly surpasses the other metrics at the end, implying that the model becomes more effective in correctly identifying coreferent mentions in the Amharic text.

### Description of results

Tables 1 and 2 presents the evaluation of the proposed Amharic coreference resolution system across standard coreference metrics: MUC**,** B^3^**,** CEAF-e**,** CEAF-m**,** and BLANC. Two experimental configurations were considered: AM-mention (evaluation of predicted mentions only) and AM-coref (full coreference resolution including mention pair linking and clustering). The AM-mention results show uniformly high F1 scores of 90.9% across all metrics, indicating that the model is highly accurate in identifying mentions in the text. This is expected as mention detection is a relatively easier task compared to full coreference resolution.

**Table 1:** Result of the proposed system.

Metric	Score (%)
MUC	72.4
B^3^	74.1
CEAF-e	69.8
CEAF-m	71.6
BLANC	70.3

**Table 2:** Experiment result.

Metric	Precision	Recall	F1
MUC	73.5	71.4	72.4
B^3^	75.2	73.1	74.1
CEAF-e	70.1	69.5	69.8
CEAF-m	72.3	70.9	71.6
BLANC	71.2	69.5	70.3

In the AM-coref setting, F1 scores are slightly lower, with values ranging from 80% (MUC F1) to 90.9% (CEAF-m F1). The decrease in performance reflects the additional complexity of correctly linking mentions to their antecedents. Among the metrics, CEAF-m F1 achieves the highest value (90.9%), highlighting the model’s ability to accurately identify coreferent entities at the cluster level. BLANC F1 (81.7%) measures the model’s overall ability to distinguish coreferent from non-coreferent pairs, revealing that some difficult mention pairs still pose challenges.

These results demonstrate that the proposed integration of contextual embeddings from mBERT with manually engineered features (mention distance, exact string match, and mention length) effectively captures both deep contextual and explicit linguistic cues. The system performs robustly even in the low-resource Amharic language setting, providing a strong baseline for future coreference resolution research.

Key Insights:

- High mention detection accuracy indicates reliable preprocessing and feature extraction.
- Slightly lower coreference F1 scores point to potential improvements in handling long-distance or ambiguous mentions.
- The combination of semantic (mBERT) and structural (manual) features contributes significantly to robust performance.

We conducted experiments on Amharic coreference resolution, as presented in the results above. The experimental process began with the collection and preparation of an Amharic text dataset specifically designed for coreference resolution. Following data collection, a manually annotated corpus was constructed. Feature extraction was then performed on detected mentions, and word embeddings were generated to capture both semantic and syntactic relationships between words^6,35^. Using this setup, we evaluated a BERT-based Amharic coreference resolution system, which achieved promising performance. The model obtained 81.87% F1-score, 80.46% precision, and 83.44% recall, indicating that contextualized representations are effective for capturing coreference relations in Amharic text. For comparison, Yaregal Assabie and Temesgen Dawit evaluated an Amharic anaphora resolution system using a tenfold cross-validation approach^11^. Based on their dataset, they reported a success rate of 81.79% for resolving hidden anaphors and an accuracy of 70.91% for resolving independent anaphors. While their work focuses on anaphora resolution, our study addresses the broader task of coreference resolution, demonstrating the feasibility of transformer-based models in this low-resource language setting.

## Conclusions and future work

Coreference resolution is the process of identifying and linking expressions in a text that refer to the same real-world entity. It plays an important role in many Natural Language Processing (NLP) applications, such as machine translation, question answering, and text summarization.

In this study, we proposed a transformer-based approach using BERT for Amharic coreference resolution. The system includes both training and testing phases, with components for preprocessing, contextual embedding, feature extraction, and mention prediction. By leveraging BERT, the model captures contextual information from both directions, allowing it to better understand relationships between words in Amharic text.

The experimental results show that the proposed model performs well across standard evaluation metrics, achieving F-scores of 80%, 85.71%, 90.9%, 88.86%, and 81.7% for MUC, B^3^, BLANC, CEAF-m, and CEAF-e, respectively. These results indicate that transformer-based approaches can be effectively applied to coreference resolution in low-resource languages such as Amharic.

However, the findings should be interpreted with some caution. Coreference resolution remains a challenging task that requires understanding at multiple linguistic levels, including morphology, syntax, and semantics. In addition, transformer-based models typically perform better with large annotated datasets, which are still limited for Amharic.

Despite these promising results, coreference resolution remains a highly complex task that requires linguistic knowledge at multiple levels, including morphological, syntactic, semantic, and world knowledge. Incorporating all such knowledge sources is time-consuming and computationally demanding. Consequently, this study focused on exploiting the strengths of BERT-based contextual representations to model coreference in Amharic. However, transformer-based models typically require large amounts of annotated training data to achieve optimal performance. The relatively limited size of available Amharic datasets may therefore constrain the accuracy of the proposed model.

To further improve performance, we suggest the following directions for future work:

- Incorporating syntactic parse trees to reduce noisy mention spans and enable the identification of long-distance coreference relations, potentially spanning multiple sentences or entire documents.
- Developing POS-tagged and chunked Amharic corpora, which are currently scarce, to support richer linguistic feature extraction and improve model performance.

## Supplementary Information

Supplementary Information 1.

Supplementary Information 2.

## Data Availability

The datasets used and/or analyzed during the current study available from the corresponding author on reasonable request through email address (lingerewbantie@dbu.edu.et).

## Code availability

The custom code and algorithms used in this study are publicly available at: https://github.com/Lingerew-10/Computer-Science-. The repository includes all scripts required for data preprocessing, model training, evaluation, and result reproduction. The code is maintained under version control to ensure transparency and reproducibility of the proposed approach.
